# Sport- and Side-Specific Postural-Control Profiles in Elite Athletes: A Cross-Sectional Analysis of Centre of Pressure Path Length, ML/AP Directionality, and Frequency-Domain Descriptors

**DOI:** 10.3390/sports14070303

**Published:** 2026-07-16

**Authors:** Philipp Floessel, Jan Jens Koltermann, Freya Charlotte Wunderlich, Jil-Justin Funke, Chantal Freudenberg, Alexander C. Disch

**Affiliations:** 1 University Center for Orthopaedics, Trauma and Plastic Surgery—Section Sports Medicine and Rehabilitation, Faculty of Medicine Carl Gustav Carus, Technische Universitaet Dresden, Fetscherstrasse 74, 01307 Dresden, Germany; jan.koltermann@mailbox.tu-dresden.de (J.J.K.); charlywunder@yahoo.de (F.C.W.); jil-justin.funke@ukdd.de (J.-J.F.); chantal.freudenberg@ukdd.de (C.F.);; 2 University Center for Orthopaedics, Trauma and Plastic Surgery—University Comprehensive Spine Center (UCSC), Faculty of Medicine Carl Gustav Carus, Technische Universitaet Dresden, Fetscherstrasse 74, 01307 Dresden, Germany

**Keywords:** postural control, Centre of Pressure, elite athletes, balance, power spectral density, ML/AP directionality, single-leg stance, biomechanics, frequency-domain analysis

## Abstract

Background/Objectives: Postural control in elite athletes may reflect sport- and side-related balance demands. Conventional Centre of Pressure (CoP) path length alone offers only limited information about directional and frequency-domain sway characteristics. This cross-sectional study described CoP path length, mediolateral/anteroposterior (ML/AP) directionality, and power spectral density (PSD)-derived frequency-domain descriptors in elite athletes from sports with distinct movement demands. Methods: A total of 116 asymptomatic elite athletes from volleyball, football, short track, ice hockey, and field hockey were assessed during single-leg stance. CoP path length, the ML/AP index, and PSD outcomes were analysed. PSD was calculated in LabVIEW using a fast Fourier transform (FFT) routine from the complete 60 s trial acquired at 1 kHz after removal of the DC component. Spectra were not normalised and are reported as absolute spectral-density values in mm^2^/Hz. PSD outcomes were summarised in low-frequency (0.02–0.6 Hz) and higher-frequency (1–5 Hz) windows, and the PSD quotient was defined as PSD 0.02–0.6 Hz/PSD 1–5 Hz. Results: Observed sport–sex groups differed in subject-averaged CoP path length (F(5,110) = 22.26, *p* < 0.001, eta_*p*^2^ = 0.503), ML/AP index (F(5,110) = 4.07, *p* = 0.002, eta_*p*^2^ = 0.156), PSD 0.02–0.6 Hz (F(5,110) = 38.67, *p* < 0.001, eta_*p*^2^ = 0.637), PSD 1–5 Hz (F(5,110) = 4.83, *p* < 0.001, eta_*p*^2^ = 0.180), and the exploratory PSD quotient (F(5,110) = 3.33, *p* = 0.008, eta_*p*^2^ = 0.132). Paired-side comparisons showed greater right-side CoP path length, greater right-side PSD 0.02–0.6 Hz, and a higher right-side ML/AP index, whereas PSD 1–5 Hz and the PSD quotient did not differ significantly between sides. Conclusions: The combined analysis of CoP path length, ML/AP directionality, and PSD-derived descriptors characterised sport-, sex-, and side-specific postural-control profiles in this cohort. Mechanistic interpretations of segmental neuromuscular control remain tentative because the study was cross-sectional and did not include electromyography, kinematics, or prospective injury data.

## 1. Introduction

Postural control is central to kinesiology, sports biomechanics, and athlete monitoring. It depends on the integration of musculoskeletal and neural mechanisms with visual, vestibular and somatosensory information [1,2]. Postural-control performance is influenced by anthropometry, body composition, and the functional capacity of the lower limbs and trunk musculature [3,4,5,6,7]. In elite athletes, this interaction is not merely a generic balance function. It is also embedded in the postural constraints imposed by each discipline.

Different sports impose specific constraints on support surface, preferred limb use, acceleration, rotation, and coordination between proximal and distal segments. Repeated exposure to these constraints may be associated with discipline-specific postural profiles, although cross-sectional data cannot establish training effects [8,9,10,11,12]. Such profiles may be relevant for sport-specific performance [8,10,11], and athletes often show better postural control than non-athletes [13,14]. Strategies for maintaining postural stability may also vary by sport, sex, limb function, and individual characteristics [4,8,15,16].

Athlete-focused studies indicate that impaired postural control may be associated with a higher risk of lower-limb injury [17,18]. Hallen et al. [19] reported thigh muscle injuries as the most common injury type in a large prospective observational study of European elite women’s football, whereas anterior cruciate ligament ruptures produced the greatest injury burden. Sex-specific risk profiles are also relevant because neuromuscular control may be modulated by anatomical, hormonal, and functional factors. Together with the review findings of Hamed-Hamed et al. [20] and recent work on cycle-dependent variation in neuromuscular control [21,22], these findings underline the importance of interpreting postural control within a sport- and athlete-specific framework.

One widely used approach for quantifying postural control is Centre of Pressure (CoP) analysis during quiet standing [4,8]. CoP path length reflects the spatial extent of sway, whereas decomposition into anteroposterior and mediolateral components can provide additional direction-specific information [23,24,25]. Frequency-domain analysis can also provide insight into how the spectral density of the CoP is distributed across predefined frequency windows. Previously published studies have associated low-frequency ranges (0.02 to 0.6 Hz) with trunk-related components, whereas frequencies between 1 and 5 Hz have been used to represent the contribution of the lower extremities to postural stability [26,27,28,29].

The aim of this study was therefore to describe sport- and side-related postural-control profiles in observed sport–sex groups of elite athletes by combining CoP path length, ML/AP directionality, and PSD-derived frequency-domain descriptors. We hypothesised that observed sport–sex groups would differ in CoP path length, ML/AP index, and PSD-derived low- and higher-frequency components, and that side-specific differences would be present in selected outcomes.

## 2. Materials and Methods

The investigation was designed as a cross-sectional observational study embedded in performance-diagnostic squad testing. Data were collected in 2024 during the competitive season as part of aptitude and performance diagnostics for national-team athletes at an institution certified by the German Olympic Sports Confederation. The design permits description of between-group and side-related postural-control profiles; it does not allow causal conclusions about training effects. Ethical approval was obtained from the responsible ethics committee (approval number: BO-EK-74022021_1). Before testing, all athletes were informed about the purpose and procedures of the investigation and provided written consent for participation and for the scientific analysis of anonymised data.

Asymptomatic elite athletes from volleyball, football, short track, ice hockey, and field hockey were included. The final analytical sample comprised 116 athletes, including 49 women and 67 men. The observed sport–sex groups were as follows: volleyball women, *n* = 34; football men, *n* = 19; short track men, *n* = 11; ice hockey men, *n* = 14; field hockey women, *n* = 15; and field hockey men, *n* = 23. Inclusion required an average training volume of at least 15 h per week. Exclusion criteria were acute lower-limb injuries, relevant previous surgery, and acute or chronic low back pain during the three months preceding the assessment. Back pain was recorded using the Chronic Pain Grade Questionnaire according to von Korff, for which the German version has been validated [30]. Since some sports were represented by only one sex, the effects of sport type and sex could not be fully disentangled in the primary between-group analyses. Consequently, observed differences between sports cannot be unequivocally attributed to sport-specific demands; instead, they could also stem from biological, sex-related differences in postural control.

Postural control was assessed using CoP during quiet single-leg stance. Each side was measured once for 60 s, with a 10 s rest between measurements. Athletes performed the tests barefoot, with eyes open and hands placed on the hips. They fixated a visual target positioned at a height of 1.7 m and a distance of 4 m. Foot position was standardised using visual markings; during single-leg stance, the contralateral leg was not permitted to touch the stance leg. The sequence of left and right single-leg stance was randomised. Participants were instructed to maintain an upright and stable trunk posture throughout the measurement. Because the assessment was embedded in squad diagnostic testing, repeated trials were not available for reliability analyses. Before the actual test began, the athletes performed a 10 s trial measurement for each test condition to familiarise themselves with the upcoming test scenario.

CoP data were acquired with a modified Wii Balance Board/HUMAC Balance System setup. The underlying board system and its validation against conventional force plates have been described previously [31]. That validation supports the use of the board system for CoP measurement in general. It does not, by itself, validate the present 1 kHz LabVIEW acquisition configuration or the 14-bit study-specific acquisition chain. The exported analysis archive documented acquisition frequency and resolution, but not the complete calibration constants or zeroing protocol; these technical details are therefore reported as limitations.

The CoP time series were preprocessed before outcome calculation. CoP path length in centimetres served as the primary spatial parameter. Automated and visual plausibility checks were performed. No anomalies were detected during this plausibility review; consequently, no trials were excluded. These checks did not reduce the analytical sample. Filtering was performed using a second-order Butterworth low-pass filter. The cut-off frequency was calculated according to the PSD-based procedure for determining relevant CoP frequency components described by Koltermann et al. [32]. For the present dataset, the calculated cut-off was 14.8 Hz; therefore, a fixed cut-off frequency of 15 Hz was used for subsequent analyses.

For the direction-specific description of CoP sway, we calculated an ML/AP index. The index was defined as the ratio of mediolateral to anteroposterior directional CoP movement; higher values indicate a larger relative mediolateral sway component. PSD of the CoP time series was calculated in LabVIEW with an FFT-based Power Spectrum/PSD routine. For each side, the complete 60 s trial was analysed as one segment. At 1 kHz, this corresponded to N = 60,000 samples and a frequency resolution of 0.0167 Hz. Before spectral analysis, the DC component was removed by subtracting the mean CoP value. No Welch segmentation, segment averaging, overlap correction, or signal normalisation was applied. The available LabVIEW export did not document an additional window-function setting or the exact one-sided/two-sided PSD scaling convention. These settings were therefore not inferred retrospectively and are reported as technical limitations. PSD values are presented as exported absolute spectral-density values in mm^2^/Hz. PSD outcomes were summarised in two predefined frequency windows: 0.02–0.6 Hz as a low-frequency CoP component and 1–5 Hz as a higher-frequency CoP component. These windows are indirect frequency-domain descriptors of CoP sway. Any physiological interpretation in terms of segmental or neuromuscular control should therefore be treated as hypothesis-generating. The PSD quotient was defined as PSD 0.02–0.6 Hz/PSD 1–5 Hz and is dimensionless.

Statistical analysis was performed descriptively and inferentially. Continuous variables were reported as mean and standard deviation. For between-group models, left and right values were combined into subject-level means. Because most sports were represented predominantly by one sex and a full sport-by-sex model was not of full rank, the observed sport–sex groups were modelled as the primary grouping factor. Linear models of the form outcome ~ sport–sex group were calculated for CoP path length, ML/AP index, PSD 0.02–0.6 Hz, PSD 1–5 Hz, and the exploratory PSD quotient; models were evaluated using Analysis of Variance (ANOVA), and partial eta squared was reported as the effect size. Sensitivity models added one anthropometric covariate at a time (age, height, body mass, or body mass index (BMI)). Model assumptions were examined using residual diagnostics and formal checks of residual normality and variance homogeneity; because several PSD-derived outcomes showed deviations from ideal assumptions, Welch ANOVA and Kruskal–Wallis tests were additionally calculated as robustness checks. Left–right differences were examined using paired t-tests and are reported with t-values, degrees of freedom, 95% confidence intervals, Holm-adjusted *p*-values, and paired Cohen’s dz. Associations between outcome parameters and anthropometry were described using Pearson correlations. For the field hockey cohort, in which both sexes were represented, supplementary Welch t-tests were calculated for sex comparisons. The significance level was set at alpha = 0.05. Because multiple outcomes and exploratory side comparisons were analysed, *p*-values were interpreted with attention to the exploratory character of the study. Analyses were conducted in R 4.5.2; the principal packages used were dplyr 1.2.0, tidyr 1.3.2, tibble 3.3.1, ggplot2 4.0.2, scales 1.4.0, and grid 4.5.2.

## 3. Results

### 3.1. Cohort Analysis

A total of 116 asymptomatic athletes (42.2% female) participated in this cross-sectional study. The mean age was 20.4 +/− 4.2 years, mean height was 180.4 +/− 7.9 cm, mean body mass was 74.1 +/− 10.2 kg, and mean BMI was 22.7 +/− 2.1 kg/m^2^ (Table 1). The cohort was intentionally analysed as observed sport–sex groups because several sports were represented by only one sex.

### 3.2. Spatial, Directional, and Frequency-Domain Outcomes

The observed sport–sex group factor was significant for all subject-averaged postural-control outcomes. Group effects were observed for CoP path length (F(5,110) = 22.26, *p* < 0.001, eta_*p*^2^ = 0.503), ML/AP index (F(5,110) = 4.07, *p* = 0.002, eta_*p*^2^ = 0.156), PSD 0.02–0.6 Hz (F(5,110) = 38.67, *p* < 0.001, eta_*p*^2^ = 0.637), PSD 1–5 Hz (F(5,110) = 4.83, *p* < 0.001, eta_*p*^2^ = 0.180), and the exploratory PSD quotient (F(5,110) = 3.33, *p* = 0.008, eta_*p*^2^ = 0.132). These results show group-level differences in spatial sway magnitude as well as directional and frequency-domain CoP descriptors. Importantly, these pairwise comparisons reflect the asymmetry observed across the total cohort and may not systematically generalise to every specific sport-by-sex subgroup.

Descriptive left- and right-side values are reported in Table 2 and Table 3. The group effects remained present in the one-covariate sensitivity models and robustness checks (Table 4). Paired-side comparisons across the whole cohort showed greater right-side CoP path length (mean difference: 48.57 cm, 95% CI 28.15 to 68.99; t(115) = 4.71, *p* < 0.001, dz = 0.44), greater right-side PSD 0.02–0.6 Hz (mean difference: 0.79 mm^2^/Hz, 95% CI 0.28 to 1.30; t(115) = 3.05, *p* = 0.0029, dz = 0.28), and a higher right-side ML/AP index (mean difference: 0.335, 95% CI 0.181 to 0.488; t(114) = 4.32, *p* < 0.001, dz = 0.40). PSD 1–5 Hz did not differ significantly between sides (mean difference: 0.014 mm^2^/Hz, 95% CI −0.049 to 0.077; t(115) = 0.43, *p* = 0.666, dz = 0.04). The explicitly calculated PSD quotient likewise showed no significant side difference (mean difference: 2.95, 95% CI −10.07 to 15.97; t(115) = 0.45, *p* = 0.654, dz = 0.04). Exact inferential statistics are summarised in Table 5.

### 3.3. ML/AP Directionality of CoP Movement

The ML/AP index provided direction-specific information that was not captured by CoP path length alone. Several groups showed side-related differences in the relative mediolateral sway component. These patterns are descriptive. They should be considered together with the group-level ANOVA because the sample structure does not allow independent estimation of sport, sex, and limb-dominance effects.

### 3.4. Descriptive Relationship Between CoP Path Length and ML/AP Directionality

**Figure 1:**
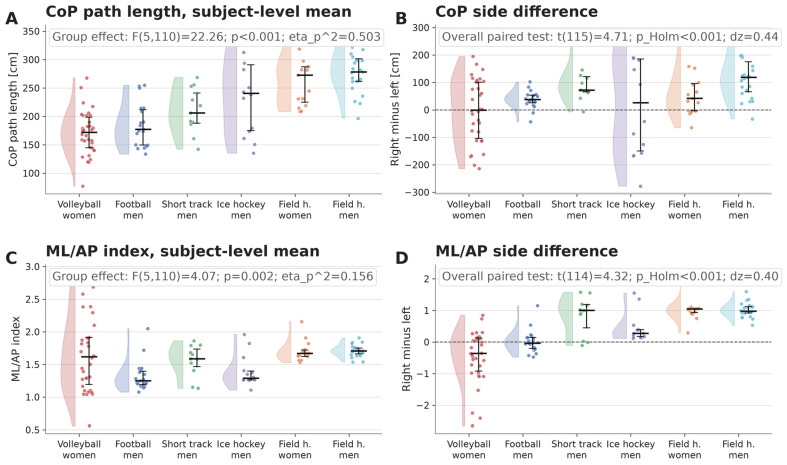
Spatial and directional CoP profiles by observed sport–sex group. Raincloud plots show the distribution, individual athletes, and median/IQR for subject-level CoP path length (**A**), CoP side difference (**B**), ML/AP index (**C**), and ML/AP side difference (**D**). Side-difference panels show right minus left; the dashed horizontal line indicates no side difference. In the x-axis labels, Field h. denotes field hockey.

Descriptively, CoP path length and ML/AP directionality did not change uniformly across groups or sides. For example, some groups showed relatively low CoP path length but pronounced ML/AP asymmetry, whereas others showed larger CoP path-length differences without a comparable directional shift. Because no formal regression model between CoP path length and ML/AP index was prespecified, this section is presented as a descriptive comparison rather than evidence of a causal or mechanistic relationship. The group- and side-specific distributions are shown in Figure 1.

Sensitivity models were calculated to examine whether the observed sport–sex group effects persisted after separate adjustment for age, height, body mass, or BMI. Across outcomes, the observed sport–sex group effect remained significant. Body mass and BMI were relevant covariates in the ML/AP model, and height was relevant in the exploratory PSD-quotient model. These findings indicate that anthropometry did not remove the observed group structure, while reinforcing the need for cautious interpretation.

### 3.5. Group-Level Inferential Statistics for CoP and PSD Outcomes

Table 5 summarises the group-level inferential statistics and the paired-side comparisons. The table reports F-values, t-values, degrees of freedom, *p*-values, Holm-adjusted side-comparison *p*-values, confidence intervals, and effect sizes. PSD outcomes are reported as CoP spectral-density descriptors rather than as measures of energetic demand.

### 3.6. Low-Frequency PSD Component (0.02–0.6 Hz)

The low-frequency PSD component differed between observed sport–sex groups (F(5,110) = 38.67, *p* < 0.001, eta_*p*^2^ = 0.637). It was also greater on the right side in the paired whole-cohort comparison (mean difference: 0.79 mm^2^/Hz, 95% CI 0.28 to 1.30; t(115) = 3.05, *p* = 0.0029, Holm-adjusted *p* = 0.0086). These values describe low-frequency CoP spectral density in mm^2^/Hz. Any interpretation as segmental neuromuscular control remains an indirect physiological hypothesis.

Table 6 shows the means and standard deviations of PSD values in the frequency range from 0.02 to 0.6 Hz during left and right single-leg stance, categorised by sport–sex group. Values are absolute PSD values in mm^2^/Hz.

### 3.7. Higher-Frequency PSD Component (1–5 Hz)

The higher-frequency PSD component (1–5 Hz) differed between observed sport–sex groups (F(5,110) = 4.83, *p* < 0.001, eta_*p*^2^ = 0.180), but no significant whole-cohort side difference was observed (mean difference: 0.014 mm^2^/Hz, 95% CI −0.049 to 0.077; t(115) = 0.43, *p* = 0.666). This band is therefore reported as a higher-frequency CoP spectral descriptor. Direct claims about distal neuromuscular control require validation with kinematic, electromyographic or inertial-sensor data.

Table 7 shows the means and standard deviations of PSD values in the frequency range from 1 to 5 Hz during left and right single-leg stance, categorised by sport–sex group. Values are absolute PSD values in mm^2^/Hz.

### 3.8. PSD Quotient and Consolidated Frequency-Domain Interpretation

The PSD quotient was reported consistently as PSD 0.02–0.6 Hz/PSD 1–5 Hz. The quotient is dimensionless and was analysed as an exploratory descriptor of the relative balance between low- and higher-frequency CoP components. Its mechanistic interpretation remains cautious because the quotient is sensitive to small denominator values and was not validated against direct measures of segmental muscle activity.

**Figure 2:**
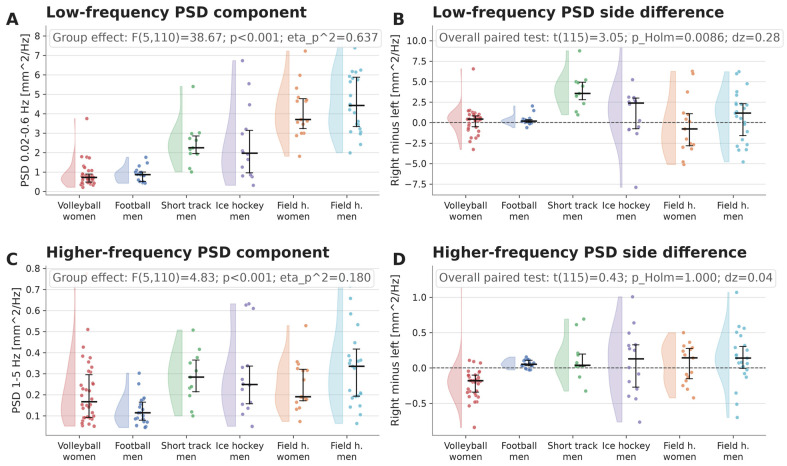
Frequency-domain CoP descriptors by observed sport–sex group. Raincloud plots show the distribution, individual athletes, and median/IQR for the low-frequency PSD component (0.02–0.6 Hz; (**A**)), low-frequency PSD side difference (**B**), higher-frequency PSD component (1–5 Hz; (**C**)), and higher-frequency PSD side difference (**D**). PSD values are exported as absolute spectral-density descriptors in mm^2^/Hz; side-difference panels show right minus left and the dashed horizontal line indicates no side difference. In the x-axis labels, Field h. denotes field hockey.

Table 8 therefore reports the exploratory PSD quotient by observed sport–sex group and side. Percentage-based PSD descriptors are not included in the main table because their denominator and interpretation were insufficiently documented and could be mistaken for mutually exclusive shares of total spectral power. Figure 2 displays the distributions of the low- and higher-frequency PSD components and their side differences.

## 4. Discussion

The present cross-sectional study examined sport- and side-related postural-control profiles in elite athletes by combining CoP path length, ML/AP directionality, and PSD-derived frequency-domain descriptors. The principal finding is that the observed sport–sex groups differed across spatial, directional, and frequency-domain outcomes. This supports the value of a multidimensional CoP assessment, while avoiding the stronger claim that PSD-derived variables identify subsystem-specific neuromuscular mechanisms.

The observed between-group differences are consistent with the view that postural control may be associated with sport-specific task constraints. However, these findings should be framed as group-specific profiles rather than causal training effects. The present design cannot determine whether the observed profiles resulted from sport-specific training, athlete selection, sex-related factors, anthropometry, limb dominance, or unmeasured injury history.

The divergent descriptive patterns in volleyball and field hockey may be interpreted in light of their different movement demands, but such explanations remain hypotheses. It must also be emphasised that the supplementary comparison with field hockey offers merely a contrasting description and does not constitute a methodological solution to the broader problem of these confounding variables. Volleyball includes repeated jumping and landing tasks, whereas field hockey includes low-position acceleration and asymmetric stick-handling constraints. These sport-biomechanical considerations may help formulate future hypotheses, but they were not directly measured in the present study. Previous studies in volleyball and field hockey have also reported sport- and injury-related balance or performance characteristics [33,34,35].

The confounding of sport and sex is a central interpretative limitation. Volleyball was represented only by women, football, short track and ice hockey only by men, and both sexes were available only in field hockey. The supplementary field hockey comparison showed clear anthropometric sex differences but no significant sex differences in the analysed postural-control outcomes. This result is informative, but it cannot resolve the broader confounding structure of the whole sample. Comparable sex- and sport-related balance differences have also been reported in athlete populations [12,36].

The ML/AP findings suggest that CoP path length alone may not fully describe the organisation of single-leg stance. Higher ML/AP values indicate a larger relative mediolateral sway component, but they should not be equated directly with hip-dominant or ankle-dominant control without joint kinematics. The directional index is therefore best interpreted as an additional descriptive CoP parameter. This cautious interpretation is consistent with models that integrate ankle and hip contributions to stance control [37].

The football findings would require leg-dominance data for a robust interpretation in terms of kicking-leg and stance-leg demands. Because preferred kicking leg or functional dominance was not available, dominance-related explanations remain speculative. Studies comparing preferred and nonpreferred lower limbs likewise indicate that dominance can affect single-leg balance-related measures [38,39,40,41].

The short track and ice hockey patterns are biomechanically plausible because skating sports involve narrow support surfaces and high mediolateral control demands. Nevertheless, the present barefoot static single-leg test does not reproduce skating mechanics. The findings should therefore be interpreted as possible residual postural-control profiles rather than direct evidence of persistent skating-specific motor effects. Related work in ice hockey has described postural-sway dynamics in relation to training history [42].

The PSD results add frequency-domain information to conventional CoP path length. Higher values in the 0.02–0.6 Hz and 1–5 Hz windows describe different frequency components of CoP sway. Any attribution of these bands to segmental neuromuscular mechanisms should be phrased as an indirect interpretation based on previous models, because no electromyography, kinematics, or inertial-sensor validation was included. In line with this, the wide confidence intervals observed for the PSD quotient underline its statistical instability. Because ratio-based metrics disproportionately amplify small variances when denominator values are low, we refrain from assigning excessive interpretative weight to this quotient. Instead, it should be viewed as an exploratory indicator rather than a robust standalone diagnostic tool.

From an applied perspective, combined CoP, ML/AP, and PSD descriptors may be useful for future athlete monitoring. However, the current data do not justify specific training prescriptions or injury-prevention thresholds. Such applications require prospective studies, repeated measurements, and validation against performance, injury, and neuromuscular outcomes.

Compared with CoP path length alone, the multidimensional approach may help distinguish athletes or groups with similar spatial sway magnitude but different directional or frequency-domain profiles. It is therefore best presented as a measurement and hypothesis-generating advantage rather than as proof of distinct compensatory neuromuscular strategies.

Several limitations require consideration. First, the cross-sectional design precludes causal conclusions about training effects. Second, the observed groups were unbalanced; in particular, the short-track subgroup was very small, thereby limiting the reliability of all estimates for this group. Furthermore, the factor of sport was strongly confounded with sex. Between-group differences therefore cannot be attributed uniquely to sport-specific demands. Third, each condition was measured once, so trial-to-trial reliability and familiarisation effects could not be quantified.

Fourth, leg dominance, preferred kicking or stance leg, previous injury history, and menstrual-cycle phase were not available for all athletes. Fifth, no electromyography, kinematic, or inertial-sensor data were collected. Low- and higher-frequency PSD components therefore cannot be interpreted as direct measures of segmental neuromuscular control.

Sixth, the measurements used modified Wii Balance Board/HUMAC technology. Although this technology has been validated against force plates [31], the 1 kHz LabVIEW acquisition configuration, exact calibration constants, zeroing protocol, window-function setting, and exact PSD scaling convention of the exported LabVIEW routine could not be fully reconstructed from the available analysis files. Finally, multiple outcomes and exploratory comparisons were analysed; the inferential results should therefore be interpreted cautiously and confirmed in prospective, adequately powered studies.

## 5. Conclusions

Elite athletes from different observed sport–sex groups showed distinguishable postural-control profiles when CoP path length was analysed together with ML/AP directionality and PSD-derived frequency-domain descriptors. The findings support the complementary use of spatial, directional, and spectral CoP parameters in elite-athlete assessment. However, PSD-derived variables should be understood as indirect descriptors of sway frequency content rather than direct evidence of segmental neuromuscular control. Longitudinal studies with repeated trials, dominance information, electromyography, kinematics, and prospective injury or performance outcomes are required before discipline-specific training recommendations or clinical thresholds can be derived.

## Figures and Tables

**Table 1:** Anthropometric characteristics by sex and sport.

Sex	Sport	n	Age [years]	Height [cm]	Body Mass [kg]	BMI [kg/m^2^]
Women	Volleyball	34	17.22 ± 1.29	183.88 ± 7.45	72.16 ± 9.67	21.26 ± 1.87
Women	Field hockey	15	21.27 ± 3.77	170.47 ± 5.45	63.40 ± 7.07	21.76 ± 1.51
Men	Football	19	23.56 ± 4.08	182.58 ± 6.90	78.73 ± 7.58	23.56 ± 0.95
Men	Short track	11	17.65 ± 0.49	174.73 ± 6.51	67.47 ± 4.29	22.12 ± 1.30
Men	Ice hockey	14	23.59 ± 3.91	181.43 ± 4.47	84.79 ± 5.95	25.75 ± 1.30
Men	Field hockey	23	21.39 ± 4.52	182.00 ± 6.98	76.74 ± 9.60	23.07 ± 1.69

**Table 2:** CoP path length during left and right single-leg stance, presented as mean and standard deviation.

Sport	CoP Left [cm]	CoP Right [cm]
Volleyball women	169.65 ± 72.46	172.10 ± 65.99
Football men	204.54 ± 39.07	166.22 ± 44.24
Short track men	256.95 ± 58.14	161.79 ± 44.64
Ice hockey men	248.74 ± 88.35	226.94 ± 88.94
Field hockey women	236.36 ± 41.62	306.70 ± 79.00
Field hockey men	291.39 ± 74.95	233.14 ± 36.49

**Table 3:** ML/AP ratio during left and right single-leg stance, presented as mean and standard deviation.

Sport	ML/AP Left	ML/AP Right
Volleyball women	1.44 ± 0.61	1.90 ± 0.81
Football men	1.34 ± 0.35	1.31 ± 0.22
Short track men	1.98 ± 0.53	1.14 ± 0.14
Ice hockey men	1.60 ± 0.45	1.20 ± 0.13
Field hockey women	1.15 ± 0.11	2.25 ± 0.34
Field hockey men	2.22 ± 0.30	1.19 ± 0.19

**Table 4:** Sensitivity models for observed sport–sex group effects after one-covariate adjustment.

Outcome	Unadjusted Group Model	Adjusted Group *p* Range	Relevant Covariate Signal	Robustness Checks
CoP path length	F(5,110) = 22.26; *p* < 0.001; eta_*p*^2^ = 0.503	*p* < 0.001	none	Welch *p* < 0.001; Kruskal *p* < 0.001
PSD 0.02–0.6 Hz	F(5,110) = 38.67; *p* < 0.001; eta_*p*^2^ = 0.637	*p* < 0.001	none	Welch *p* < 0.001; Kruskal *p* < 0.001
PSD 1–5 Hz	F(5,110) = 4.83; *p* < 0.001; eta_*p*^2^ = 0.180	*p* < 0.001	none	Welch *p* < 0.001; Kruskal *p* < 0.001
ML/AP index	F(5,110) = 4.07; *p* = 0.002; eta_*p*^2^ = 0.156	*p* = 0.0013–0.0021	body mass *p* = 0.028; BMI *p* = 0.007	Welch *p* < 0.001; Kruskal *p* < 0.001
PSD quotient	F(5,110) = 3.33; *p* = 0.008; eta_*p*^2^ = 0.132	*p* = 0.0059–0.0082	height *p* = 0.017	Welch *p* = 0.007; Kruskal *p* < 0.001

**Table 5:** Group-level inferential statistics and paired-side comparisons.

Outcome	Sport–Sex Group Model	Side Difference Right–Left (95% CI)	Paired-Side Test	Effect Size
CoP path length	F(5,110) = 22.26; *p* < 0.001; eta_*p*^2^ = 0.503	48.57 cm (28.15 to 68.99)	t(115) = 4.71; *p* < 0.001; *p*_Holm < 0.001	0.44
ML/AP index	F(5,110) = 4.07; *p* = 0.002; eta_*p*^2^ = 0.156	0.335 (0.181 to 0.488)	t(114) = 4.32; *p* < 0.001; *p*_Holm < 0.001	0.40
PSD 0.02–0.6 Hz	F(5,110) = 38.67; *p* < 0.001; eta_*p*^2^ = 0.637	0.79 mm^2^/Hz (0.28 to 1.30)	t(115) = 3.05; *p* = 0.0029; *p*_Holm = 0.0086	0.28
PSD 1–5 Hz	F(5,110) = 4.83; *p* < 0.001; eta_*p*^2^ = 0.180	0.014 mm^2^/Hz (−0.049 to 0.077)	t(115) = 0.43; *p* = 0.666; *p*_Holm = 1.000	0.04
PSD quotient	F(5,110) = 3.33; *p* = 0.008; eta_*p*^2^ = 0.132	2.95 (−10.07 to 15.97)	t(115) = 0.45; *p* = 0.654; *p*_Holm = 1.000	0.04

**Table 6:** Low-frequency PSD component (0.02–0.6 Hz) during left and right single-leg stance [mm^2^/Hz].

Sport	PSD 0.02–0.6 Hz Left [mm^2^/Hz]	PSD 0.02–0.6 Hz Right [mm^2^/Hz]
Volleyball women	0.98 ± 1.15	0.74 ± 0.89
Football men	1.02 ± 0.61	0.71 ± 0.26
Short track men	4.44 ± 2.24	0.52 ± 0.22
Ice hockey men	3.23 ± 3.37	1.71 ± 2.17
Field hockey women	4.19 ± 2.21	3.78 ± 2.30
Field hockey men	3.98 ± 2.16	4.34 ± 2.33

**Table 7:** Higher-frequency PSD component (1–5 Hz) during left and right single-leg stance [mm^2^/Hz].

Sport	PSD 1–5 Hz Left [mm^2^/Hz]	PSD 1–5 Hz Right [mm^2^/Hz]
Volleyball women	0.12 ± 0.24	0.30 ± 0.21
Football men	0.16 ± 0.07	0.10 ± 0.07
Short track men	0.35 ± 0.19	0.22 ± 0.20
Ice hockey men	0.34 ± 0.34	0.26 ± 0.27
Field hockey women	0.22 ± 0.14	0.27 ± 0.22
Field hockey men	0.28 ± 0.22	0.22 ± 0.13

**Table 8:** Exploratory PSD quotient based on PSD 0.02–0.6 Hz/PSD 1–5 Hz.

Observed Sport–Sex Group	n	Left Mean	Left SD	Right Mean	Right SD	Mean Quotient	SD Quotient	Interpretation
Volleyball women	34	3.87	4.16	29.11	44.39	16.49	23.05	exploratory
Football men	19	10.45	6.53	8.72	9.46	9.59	7.49	exploratory
Short track men	11	6.88	8.48	15.95	10.07	11.42	5.95	exploratory
Ice hockey men	14	10.59	7.21	18.63	21.51	14.61	11.24	exploratory
Field hockey women	15	65.65	166.69	37.53	50.54	51.59	84.16	unstable denominator
Field hockey men	23	28.72	25.09	16.83	11.32	22.77	14.40	exploratory

## Data Availability

The datasets analysed during the current study are available from the corresponding author upon reasonable request. The data are not publicly available because they contain potentially identifiable participant information and are subject to institutional and ethical data-protection requirements.
